# Varicella-Zoster Virus Meningitis With Hypoglycorrhachia Mimicking Tuberculous Meningitis: A Case Report

**DOI:** 10.7759/cureus.83007

**Published:** 2025-04-25

**Authors:** Sho Matsushita, Yoji Hirayama, Yoriko Matsuyama, Tatsuya Aoki, Ryosuke Horitani

**Affiliations:** 1 Department of General Internal Medicine, Hashimoto Municipal Hospital, Hashimoto, JPN

**Keywords:** hypoglycorrhachia, meningitis, multiplex pcr, tuberculosis, varicella-zoster virus

## Abstract

Varicella-Zoster virus (VZV) meningitis poses diagnostic challenges and often presents with atypical clinical and laboratory findings. We present a case of VZV meningitis with hypoglycorrhachia mimicking tuberculous meningitis (TBM). A 65-year-old Japanese woman with a past history of successfully treated tuberculosis presented with a persistent headache. Cerebrospinal fluid (CSF) analysis showed lymphocytic pleocytosis and hypoglycorrhachia (CSF glucose: 43 mg/dL, CSF-to-serum glucose ratio: 0.34) with borderline elevated adenosine deaminase ADA level (9.1 IU/L), raising concern for TBM. Head computed tomography (CT) was unremarkable, and chest CT showed only calcified nodules consistent with prior tuberculous infection. The patient met the criteria for *possible* TBM based on clinical and CSF findings. Empirical treatment with broad-spectrum antibiotics and dexamethasone was initiated for suspected bacterial meningitis. Her symptoms improved rapidly, and both CSF and blood cultures were negative. A multiplex polymerase chain reaction (PCR) test later identified VZV as the causative pathogen. Antiviral therapy was withheld, as the patient’s symptoms had fully resolved by the time of diagnosis. She remained well thereafter, with no complications during follow-up. This case illustrates how VZV meningitis can mimic TBM through marked hypoglycorrhachia and a remote history of tuberculosis.

## Introduction

Varicella-Zoster virus (VZV) is a type of human herpesvirus that remains latent in nerve ganglia after causing primary varicella (chickenpox). Reactivation of latent VZV can lead to neurological complications, including meningitis, encephalitis, and vasculopathy [1]. VZV meningitis accounts for approximately 7% of meningitis cases in the United Kingdom and typically affects immunocompetent older adults, presenting with fever, headache, and cerebrospinal fluid (CSF) lymphocytic pleocytosis with normal glucose levels [2].

However, hypoglycorrhachia (low CSF glucose concentration) occurs in approximately 20% of VZV meningitis cases [3], although the mechanism by which VZV lowers CSF glucose is not fully understood [4]. This finding can mimic tuberculous meningitis (TBM), which is characterized by lymphocytic pleocytosis, elevated protein levels, and hypoglycorrhachia in over 80% of cases [5].

In this report, we present a case of VZV meningitis with hypoglycorrhachia in a patient with a remote history of pulmonary tuberculosis whose CSF profile resembled that of TBM. The diagnosis was made by multiplex polymerase chain reaction (PCR), which can detect 14 pathogens simultaneously [6]. This case highlights the diagnostic challenges in distinguishing VZV meningitis with hypoglycorrhachia from TBM and underscores the role of PCR-based testing in guiding clinical management.

## Case presentation

A 65-year-old woman presented with a persistent headache, fever, and nausea. She had been in her usual state until six days before admission, when she developed a bilateral temporal headache rated 8 out of 10 on a numerical pain scale. The headache was accompanied by fever and nausea for four days before admission. She did not experience photophobia, aura, or altered mental status. Three days before admission, she visited her primary care physician, where her headache was attributed to shoulder stiffness, and she was prescribed acetaminophen. However, the headache did not improve, and posterior neck pain progressed. One day before admission, she visited another clinic specializing in headaches, where she was prescribed ibuprofen and rizatriptan. These medications failed to relieve her symptoms, and the headache became severe enough to awaken her during the night. Despite analgesic use, the headache persisted, prompting her to visit the emergency department of our hospital. Vomiting was not present before admission but developed on the day of presentation.

The patient had a medical history of pulmonary tuberculosis approximately 30 years earlier. She reportedly received a full course of anti-tuberculosis therapy and was considered cured at the time. Although the patient had a history of tension-type headaches associated with shoulder stiffness, the current episode was markedly more severe and qualitatively different from her usual headaches. She was not taking any medications other than those prescribed at presentation, and had no prior history of chronic use of nonsteroidal anti-inflammatory drugs (NSAIDs) or triptans. Her social history included smoking 10 cigarettes per day for 20 years and alcohol consumption once a week. She had no known allergies or travel history.

On physical examination, the patient was conscious and alert. Her vital signs were as follows: body temperature, 38.1 °C; heart rate, 60 beats per minute; blood pressure, 155/75 mmHg; respiratory rate, 20 breaths per minute; and oxygen saturation, 96% on room air. Neurological findings, including neck stiffness, Kernig’s sign, and the jolt accentuation test, were negative. Physical examination revealed no notable abnormalities or rashes. Laboratory results revealed mild hyponatremia with no elevation of inflammatory markers (Table 1).

**Table 1:** Laboratory findings on admission. WBC, white blood cell; Hb, hemoglobin; Plt, platelet; TP, total protein; Alb, albumin; AST, aspartate aminotransferase; ALT, alanine aminotransferase; ALP, alkaline phosphatase; LDH, lactate dehydrogenase; BUN, blood urea nitrogen; Cre, creatinine; Na, sodium; K, potassium; Cl, chloride; Glu, glucose; CRP, C-reactive protein

Laboratory parameter	Day 1	Reference range
WBC (×10^3^/μL)	7.5	3.3-8.6
Hb (g/dL)	13.8	11.6-14.8
Plt (×10^4^/μL)	29.5	15.8-34.8
TP (g/dL)	7.5	6.6-8.1
Alb (g/dL)	4.1	4.1-5.1
AST (U/L)	24	13-30
ALT (U/L)	22	7-23
ALP (U/L)	58	38-113
LDH (U/L)	177	124-222
BUN (mg/dL)	10.2	8.0-20.0
Cre (mg/dL)	0.69	0.46-0.79
Na (mmol/L)	134	138-145
K (mmol/L)	3.9	3.6-4.8
Cl (mmol/L)	97	96-106
Glu (mg/dL)	128	70-105
CRP (mg/dL)	0.03	0.00-0.14

**Figure 1:**
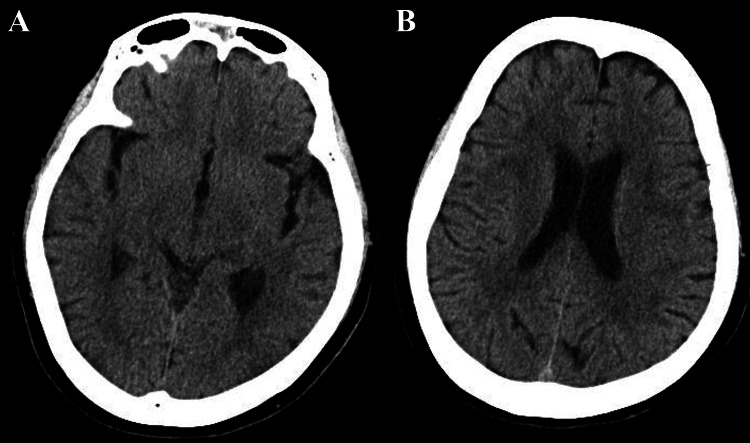
Head computed tomography (CT) revealing no hemorrhage or calcification (A, B).

After a head CT showed no evidence of increased intracranial pressure (Figure 1), a lumbar puncture revealed mononuclear cell-predominant pleocytosis and hypoglycorrhachia (Table 2). The CSF glucose was 43 mg/dL, the serum glucose was 128 mg/dL, and the CSF-to-serum glucose ratio was 0.34. CSF analysis showed 1,000 red blood cells/μL, which was attributed to a traumatic tap. CSF was submitted for bacterial and mycobacterial cultures, and blood cultures were also obtained, along with CSF testing for tuberculosis PCR, adenosine deaminase (ADA) assay, and multiplex PCR. Because multiplex PCR was not routinely performed in-house at our hospital, the specimen was sent to an external laboratory.

**Table 2:** Cerebrospinal fluid findings. †As reference values were not established at our institution, we referred to those provided by LSI Medience Corporation (https://data.medience.co.jp/guide/guide-12050003.html). ‡Similarly, as no reference range for cerebrospinal fluid ADA was established at our institution, we referred to a study that reported ADA levels of 1-4 IU/L to be useful in excluding tuberculous meningitis [7]. RBC, red blood cell; CSF, cerebrospinal fluid; ADA, adenosine deaminase; PCR, polymerase chain reaction; VZV, Varicella-Zoster virus

Cerebrospinal fluid findings	Day 1	Day 6	Reference range
Appearance	Clear	Clear	Clear
Cells (/μL)	327	467	<5
Mononuclear cell (%)	99.4	99.4	80-100†
Polynuclear cell (%)	0.6	0.6	2-3†
RBC	1,000	0	0†
Protein (mg/dL)	116	40	15-40
Glucose (mg/dL)	43	53	50-80
CSF-to-serum glucose ratio	0.34	0.63	<0.5
ADA (U/L)	9.1	6.2	1-4‡
Tuberculosis PCR	Negative	Negative	Negative
Multiplex PCR	Positive for VZV	Not tested	Negative
VZV PCR	Not tested	Positive	Negative
Bacterial culture	Negative	Negative	Negative
Mycobacterial culture	Negative	Negative	Negative

**Figure 2:**
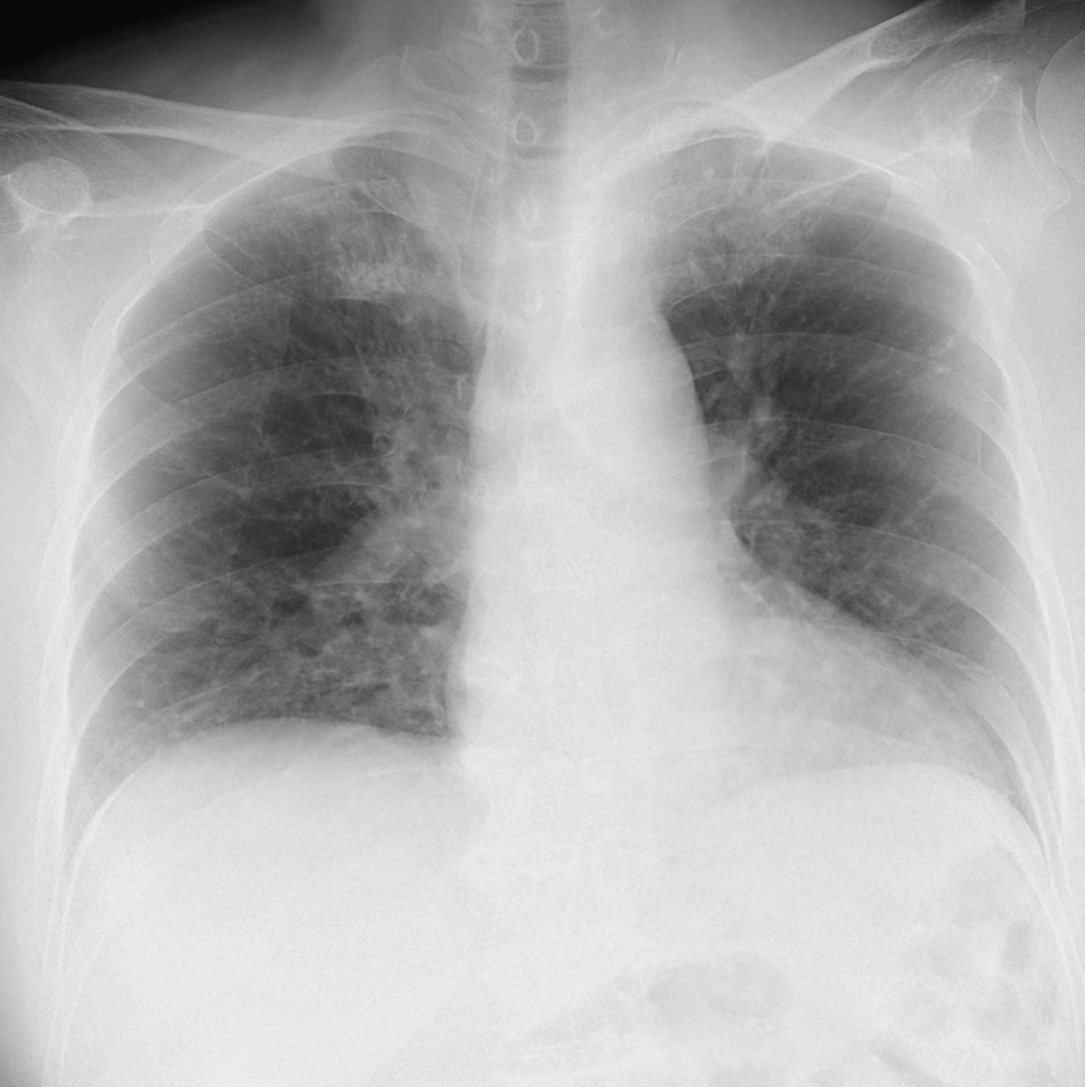
Chest X-ray in the sitting position showed no evidence of active pulmonary lesions.

**Figure 3:**
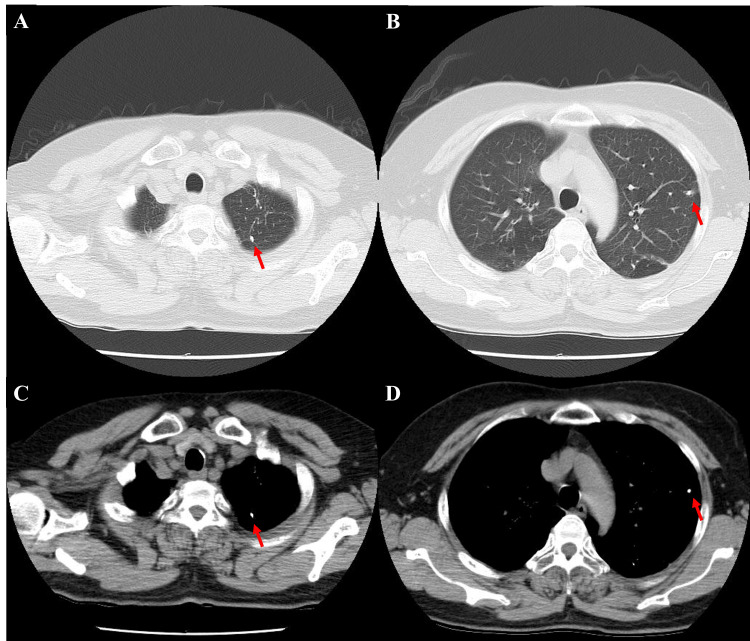
Chest computed tomography (CT) revealing nodules with calcification in the apical regions of the lungs (A, C) and the left upper lobe (B, D), consistent with old (inactive) tuberculosis.

Chest X-ray on admission showed no active pulmonary lesions (Figure 2). Chest CT revealed nodules with calcification in the apical region of the lungs and the left upper lobe, consistent with old (inactive) tuberculosis; no tree-in-bud appearance or cavitations were observed (Figure 3).

The differential diagnosis included infectious causes such as viral, bacterial, and tuberculous meningitis, as well as non-infectious etiologies like migraine and medication overuse headache (MOH). MOH was considered unlikely, as the patient had taken only small amounts of ibuprofen and rizatriptan one day before admission, without a history of chronic analgesic use. Migraine was also deemed unlikely due to the absence of photophobia, aura, or prior migraine history. Although she had a history of tension-type headaches triggered by shoulder stiffness, the current episode was markedly more severe and qualitatively different, making this diagnosis less likely.

Given the presence of lymphocytic pleocytosis and hypoglycorrhachia, infectious causes were prioritized. The lack of meningeal signs, such as neck stiffness, is consistent with previous reports that these findings may be absent in meningitis, particularly in older adults [8]. Mild, euvolemic hyponatremia suggested a syndrome of inappropriate antidiuresis (SIAD), a complication often seen in central nervous system (CNS) infections.

In particular, TBM was considered due to the patient’s remote history of pulmonary tuberculosis and CSF findings suggestive of TBM. According to the Lancet scoring system, the patient met the criteria for *possible* TBM (score of 8), based on symptom duration longer than five days, clear CSF appearance, lymphocytic predominance, and a CSF-to-serum glucose ratio <0.5 [9].

Although the clinical presentation suggested aseptic meningitis, bacterial infection could not be completely excluded. The patient was admitted and empirically started on ceftriaxone, vancomycin, ampicillin, and dexamethasone, pending the results of multiplex PCR, CSF bacterial and tuberculosis smears, cultures, and tuberculosis PCR. 

**Figure 4:**
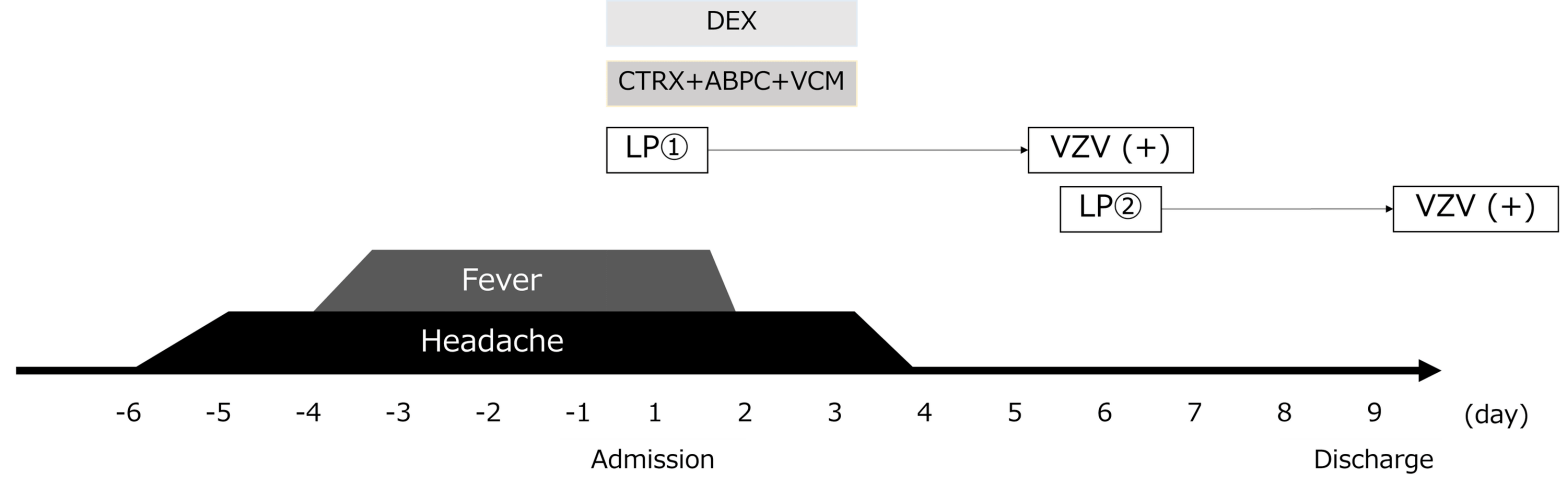
Clinical course. **On day 6, multiplex polymerase chain reaction (PCR) returned positive for VZV. A second lumbar puncture was performed to confirm the diagnosis and assess recovery from hypoglycorrhachia. DEX, dexamethasone; CTRX, ceftriaxone; ABPC, ampicillin; VCM, vancomycin; LP, lumbar puncture; VZV, Varicella-Zoster virus

The patient was afebrile on the second day of hospitalization. On day 3, antibiotics and dexamethasone were discontinued as CSF and blood cultures were negative, and the patient’s symptoms gradually improved. By day 4, the headache and nausea resolved completely (Figure 4). On day 6, the CSF multiplex PCR yielded positive results for VZV; as the patient's symptoms had improved, antiviral therapy was not initiated. A repeated lumbar puncture was performed to assess the clinical course and to order a VZV-PCR test, ensuring that the result of the multiplex PCR was not a false positive. Although CSF findings revealed persistent pleocytosis, the hypoglycorrhachia improved, with a CSF-to-serum glucose ratio of 0.63 (Table 2). The patient remained stable and was discharged on day 9. Repeated CSF VZV-PCR results were also positive, confirming the diagnosis of VZV meningitis. After discharge, both tuberculosis PCR and CSF cultures from the first and second lumbar punctures returned negative, and the patient recovered without any complications.

## Discussion

We report a case of VZV meningitis with hypoglycorrhachia that initially mimicked TBM in a patient with a remote history of pulmonary tuberculosis. This case highlights three important lessons. First, VZV meningitis should be considered even when CSF glucose levels are low. Second, multiplex PCR testing can be particularly valuable when hypoglycorrhachia is present and TBM is included in the differential diagnosis. Third, although current guidelines generally recommend antiviral therapy for confirmed VZV meningitis due to the risk of complications [10], our patient recovered without acyclovir. 

Identifying the causative pathogen in meningitis is essential, especially when hypoglycorrhachia is present, as this finding raises concern for potentially life-threatening conditions such as bacterial, tuberculous, or fungal meningitis [4,11]. Viral meningitis typically features normal CSF glucose levels; however, approximately 20% of VZV meningitis cases exhibit hypoglycorrhachia, which can cause potential diagnostic confusion [3,12,13]. In our case, differentiation from TBM was particularly challenging due to the patient's remote history of pulmonary tuberculosis, borderline elevated CSF ADA level (9.1 IU/L), and CSF findings (lymphocytic pleocytosis and low glucose). Although ADA ≥10 IU/L has approximately 85% sensitivity and 90% specificity for diagnosing TBM [14], elevated ADA levels also occur in other types of meningitis, including viral (18%) and bacterial (7%) cases, necessitating PCR confirmation for accurate diagnosis [15]. Additionally, the patient scored 8 points on the Lancet diagnostic criteria, classifying the case as *possible* TBM [9]. The absence of rash throughout our patient’s illness also obscured the diagnosis of VZV infection, making clinical differentiation even more challenging.

Multiplex PCR enables the simultaneous detection of 14 pathogens with sensitivities ranging from 70% to over 90% and a specificity of over 97% [6]. When the differential diagnosis includes a broad range of potential causative organisms, multiplex PCR is particularly valuable for identifying pathogens associated with hypoglycorrhachia, such as bacteria (e.g., *Escherichia coli*, *Haemophilus influenzae*, *Listeria monocytogenes*, *Neisseria meningitidis*, and *Streptococcus pneumoniae*), viruses (e.g., herpes simplex virus [HSV], VZV, enterovirus), and fungi (e.g., *Cryptococcus neoformans*) [6]. Because in-house testing was unavailable, the CSF sample was sent to an external laboratory, resulting in a six-day turnaround. This experience highlights the need for rapid in-house diagnostic testing for central nervous system infections, especially in cases with hypoglycorrhachia. The positive VZV result established the diagnosis and prevented unnecessary anti-tuberculous therapy.

Although most cases of viral meningitis in immunocompetent adults are self-limiting, VZV infections of the CNS are associated with a higher risk of complications. Accordingly, the Infectious Diseases Society of America (IDSA) recommends intravenous antiviral therapy for all confirmed cases of VZV CNS infection [10]. However, the routine use of antivirals in immunocompetent adults remains debated [12,16], and a recent study reported favorable outcomes in 14% of patients who did not receive antiviral treatment [17]. HSV meningitis, which is more common than VZV meningitis in immunocompetent adults, also frequently resolves spontaneously without antiviral therapy [18]. In our case, the patient became afebrile by day 2 and improved rapidly by hospital day 4. Antiviral therapy was withheld when VZV was identified on day 6. Notably, her symptoms resolved completely without acyclovir. The prognostic value of hypoglycorrhachia in VZV meningitis remains unclear. Although this finding is generally associated with more severe infections, such as bacterial meningitis, and has been linked to worse outcomes [11], its significance in viral meningitis is not well established. Our case suggests that mild hypoglycorrhachia alone may not reliably predict poor outcomes in VZV meningitis. Further studies are warranted to clarify the prognostic implications of hypoglycorrhachia in viral meningitis.

## Conclusions

We encountered a case of VZV meningitis with hypoglycorrhachia mimicking tuberculous meningitis that was confirmed by multiplex PCR, which underscores the need for rapid in-house testing. The patient recovered without antiviral therapy, highlighting the need for further studies to evaluate the prognostic value of hypoglycorrhachia in VZV meningitis.

## References

[1] Gershon AA, Breuer J, Cohen JI (20151). Varicella zoster virus infection. Nat Rev Dis Primers.

[2] McGill F, Griffiths MJ, Bonnett LJ (2018). Incidence, aetiology, and sequelae of viral meningitis in UK adults: a multicentre prospective observational cohort study. Lancet Infect Dis. Sep.

[3] Inamoto A, Taniguchi T, Fujii Y, Miyoshi S (2025181). Varicella-zoster virus meningitis with hypoglycorrhachia, presenting with painless occipital herpes zoster mimicking atopic dermatitis. BMJ Case Rep.

[4] Chow E, Troy SB (2014). The differential diagnosis of hypoglycorrhachia in adult patients. Am J Med Sci. Sep.

[5] Luo M, Wang W, Zeng Q, Luo Y, Yang H, Yang X (2018). Tuberculous meningitis diagnosis and treatment in adults: A series of 189 suspected cases. Exp Ther Med.

[6] Trujillo-Gomez J, Tsokani S, Arango-Ferreira C (2022). Biofire FilmArray Meningitis/Encephalitis panel for the aetiological diagnosis of central nervous system infections: A systematic review and diagnostic test accuracy meta-analysis. EClinicalMedicine. Feb.

[7] Tuon FF, Higashino HR, Lopes MI (2010). Adenosine deaminase and tuberculous meningitis--a systematic review with meta-analysis. Scand J Infect Dis.

[8] Thomas KE, Hasbun R, Jekel J, Quagliarello VJ (2002). The diagnostic accuracy of Kernig's sign, Brudzinski's sign, and nuchal rigidity in adults with suspected meningitis. Clin Infect Dis.

[9] Marais S, Thwaites G, Schoeman JF (2010). Tuberculous meningitis: a uniform case definition for use in clinical research. Lancet Infect Dis. Nov.

[10] Tunkel AR, Glaser CA, Bloch KC (2008). The management of encephalitis: clinical practice guidelines by the Infectious Diseases Society of America. Clin Infect Dis.

[11] Shrikanth V, Salazar L, Khoury N, Wootton S, Hasbun R (2015). Hypoglycorrhachia in adults with community-acquired meningitis: etiologies and prognostic significance. Int J Infect Dis. Oct.

[12] Spernovasilis N, Milioni A, Gialamas I, Kokorakis E, Fanti G (2018). Varicella-zoster virus meningitis with hypoglycorrhachia in a young immunocompetent adult without rash: A case report and literature review. IDCases.

[13] Habib AA, Gilden D, Schmid DS, Safdieh JE (2009). Varicella zoster virus meningitis with hypoglycorrhachia in the absence of rash in an immunocompetent woman. J Neurovirol. Apr.

[14] Prasad MK, Kumar A, Nalini N (2023156). Diagnostic Accuracy of Cerebrospinal Fluid (CSF) Adenosine Deaminase (ADA) for Tuberculous Meningitis (TBM) in Adults: A Systematic Review and Meta-Analysis. Cureus.

[15] Song J, Kim SH, Jung YR, Choe J, Kang CI, Min JH (202212). 10-Year Retrospective Review of the Etiologies for Meningitis With Elevated Adenosine Deaminase in Cerebrospinal Fluid: Etiologies Other Than TB. Front Cell Infect Microbiol.

[16] Jarrin I, Sellier P, Lopes A (2016). Etiologies and Management of Aseptic Meningitis in Patients Admitted to an Internal Medicine Department. Medicine (Baltimore). Jan.

[17] Dulin M, Chevret S, Salmona M (2024). New Insights Into the Therapeutic Management of Varicella Zoster Virus Meningitis: A Series of 123 Polymerase Chain Reaction-Confirmed Cases. Open Forum Infect Dis. Jul.

[18] Kaewpoowat Q, Salazar L, Aguilera E, Wootton SH, Hasbun R (2016). Herpes simplex and varicella zoster CNS infections: clinical presentations, treatments and outcomes. Infection.

